# Micropulse Laser Trabeculoplasty with 577 nm Wavelength at 1500 or 1000 mW for Primary Open-Angle Glaucoma: A Pilot Study

**DOI:** 10.3390/life13040982

**Published:** 2023-04-10

**Authors:** Tommaso Verdina, Matteo Gironi, Bruno Battaglia, Michele Gentile, Johanna Chester, Shaniko Kaleci, Gianluca Scatigna, Rodolfo Mastropasqua, Gian Maria Cavallini

**Affiliations:** 1 Ophthalmology Clinic, University of Modena and Reggio Emilia, Azienda Ospedaliero-Universitaria di Modena, 41122 Modena, Italy; 2 Ophthalmology Clinic, Department of Medicine and Science of Ageing, University G. D’Annunzio Chieti-Pescara, 66100 Chieti, Italy; 3 Department of Surgical, Medical, Dental and Morphological Sciences Related to Transplant, Oncology and Regenerative Medicine, University of Modena and Reggio Emilia, 41121 Modena, Italy

**Keywords:** glaucoma treatment, trabeculoplasty, open-angle glaucoma, micropulse yellow laser, micropulse laser trabeculoplasty, intraocular pressure

## Abstract

**Aim**: To investigate the efficacy and safety of micropulse laser trabeculoplasty (MLT) using a 577 nm yellow wavelength laser randomly assigned to either 1500 or 1000 mW in patients with primary open-angle glaucoma (POAG). **Methods**: A prospective, double-blinded study of POAG patients was performed in a single center. MLT treatment included a 577 nm micropulse laser (IRIDEX IQ 577^TM^, IRIDEX, Mountain View, CA, USA) to 360° of the trabecular meshwork at randomly assigned varying powers: 1500 mW in one eye (MLT 1500 group) and 1000 mW in the other (MLT 1000 group). Best-corrected visual acuity (BCVA), intraocular pressure (IOP), corneal central thickness (CCT), and endothelial cell count (ECC) were evaluated at baseline (T0), post-operative 1 h (T1), 24 h (T2), 1 month (T3), 3 months (T4), and 6 months (T5) after laser treatment. Topical medications were assessed pre-treatment and at T4. **Results**: Among the 18 eyes included, we achieved a success rate (IOP reduced > 20%) in 77% of sampled eyes. In particular, IOP reduced at T2 and T3 with both MLT 1500 and 1000 without any significant differences (IOP reduction 22.9% vs. 17.3%, respectively, MLT1500 vs. MLT1000 at T2). The IOP returned to baseline values at T4 and T5 in both groups, with a reduction in topical medications administered from 2.5 ± 1.1 to 2.0 ± 1.2 to the 1500 mW group and from 2.4 ± 1.0 to 1.9 ± 1.0 to the 1000 mW group. At 1 h post-laser treatment, a transient IOP spike was registered among the MLT1500 group. There were no differences in CCT and ECC at any timepoint according to the laser powers. **Conclusions**: Over a 6-month follow-up period, 577 nm MLT at either 1500 or 1000 mW reduces IOP, enabling a stable reduction in the number of topical medications required for patients treated for POAG without any significant difference in terms of effectiveness and safety.

## 1. Introduction

Glaucoma is a progressive optic neuropathy characterized by the loss of retinal ganglion cells and their axons, causing a characteristic alteration in the optic nerve head with corresponding alterations in the visual field. It represents the first cause of irreversible blindness in the world, affecting about 55 million individuals globally [1].

The most common form of glaucoma among Caucasian patients is primary open-angle glaucoma (POAG), defined by an open, normal-appearing anterior chamber angle with increased intraocular pressure (IOP), defined as >21 mmHg. To date, the control of IOP represents the only modifiable risk factor for delaying the progression of glaucoma and can be achieved with medical, laser, or surgical therapies.

Laser treatments for glaucoma decrease IOP by either increasing the aqueous outflow (laser trabeculoplasty) or decreasing aqueous production (laser transscleral cyclophotocoagulation). Laser trabeculoplasty has a hypotonic efficacy comparable to topical medical therapy; however, it results in a better quality of life perceived by the patient and better circadian control of IOP, especially in patients who are poorly compliant with topical medication applications and in terms of the cost–benefit ratio [2,3,4,5,6].

Argon laser trabeculoplasty (ALT), available since the 1970s, applies laser spots to the trabecular meshwork to increase the aqueous outflow and decrease IOP [7,8]. In 1974, Worthen and Wickham were the first to establish that the use of a continuous-wave argon laser could be a solution to photocoagulate the trabecular meshwork and obtain a significant reduction in IOP [9]. Subsequently, in 1979, Wise and Witter published the pilot study for ALT, in which they used a much lower laser energy, resulting in a significant lowering of IOP in patients for whom medical therapy had failed [8]. ALT was initially used as an alternative to medical therapy. Over the years, several studies confirmed the positive effects in controlling IOP and favored its diffusion. However, despite proving effective in controlling IOP, normal ocular tissue function has been found to be altered by thermal damage to the trabecular meshwork and its application was limited by several complications resulting from its use, including pressure spikes higher than 10 mmHg in 12% of patients; late spikes, especially in pseudo-exfoliative glaucoma; peripheral anterior synechiae in 15–20% of cases; and damage to the corneal endothelium [10,11].

Selective laser trabeculoplasty (SLT) is a frequency-doubled, Q-switched, 532 nm Nd:YAG laser introduced in 1995 by Latina and Park [12]. SLT uses a short laser pulse in the order of nanoseconds with a reduced energy value in order to achieve selective photo-thermolysis, which prevents the transmission of heat to the surrounding non-pigmented tissues. Moreover, we can consider the thermal relaxation time of melanin to be about one microsecond. By using pulses of less than one microsecond, energy is deposited in target cells more rapidly than this energy tends to disperse into contiguous tissues. The damage is then limited to the pigmented cells only, performing selective thermolysis, preventing the transmission of heat to the surrounding non-pigmented tissues [12,13]. The SLT results are comparable to ALT with a lower incidence of adverse effects without the scarring of the trabecular meshwork and allowing for follow-up repeatability [14,15].

According to the results of the recently published large-scale multicenter randomized study of SLT versus eye drops for the first-line treatment of glaucoma and ocular hypertension patients (LiGHT trial), the effectiveness of laser therapy was demonstrated as the first therapeutic choice for POAG treatment and numerous centers have begun to offer laser trabeculoplasty, reducing patients’ dependence on eye drops [6]. However, SLT leads to higher levels of inflammation with trabecular meshwork cellular damage and excessive early pigment dispersion causing other adverse effects (i.e., acute iritis, cystoid macular edema, transient corneal thinning, endothelial decompensation, peripheral anterior synechiae, and corneal haze) and, moreover, has a limited efficacy over time [16,17,18].

SLT complications derive from damage to the trabecular endothelial cells with the fragmentation of cytoplasmic granular pigments [19]. It has been hypothesized that the damage to the trabecular meshwork and the excessive dispersion of pigments cause high levels of inflammation, which were responsible for the possible complications identified; nevertheless, the increased water outflow is precisely determined by this inflammation, which is both responsible for the lowering of blood pressure and the resulting complications. In order to reduce the complications, several researchers attempted to reduce the amount of energy used during the SLT procedure and concluded that subthreshold SLT techniques could be comparable in terms of efficacy to the standard technique [20].

Other subthreshold laser techniques were then experimented with and, in particular, Ingvoldstad and Willoughby, in 2005, described, for the first time, micropulsed laser trabeculoplasty (MLT) [21]. MLT is a new, promising technology that, with alternating short pulses and periods of pauses, does not induce thermal cellular damage to the surrounding trabecular meshwork tissue, as the period between the micropulses allows the pigmented cells to return to a basal temperature prior to the subsequent pulse, thus avoiding post-treatment IOP spikes [19,20]. The molecular effect of MLT is still unknown and its hypotensive effect is supposedly caused by the photo-stimulation rather than photocoagulation of the trabecular meshwork; although, a biological response with cytokine mediator release has been observed [22]. Furthermore, the therapeutic efficacy of MLT for POAG seems to be comparable to standard treatments reducing IOP in patients with POAG to the same degree as ALT, whilst avoiding both thermal and cellular damage (the main complications, respectively, of ALT and SLT) [21]. However, the available data comparing MLT and SLT are limited and only few studies, mostly limited to 810 nm lasers, assess its effects on IOP according to laser parameters and the duration of any eventual improvements [23]. At present, there are no gold standard indications for the optimal power value in 577 nm MLT for POAG, and the absence of a visible effect on trabecular meshwork tissues makes the titration of energy difficult and no standard protocol for MLT treatment has been defined yet. At present, the most commonly used power for both MLT with a 577 nm yellow wavelength and MLT with a 532 nm green wavelength is 1000 mW; however, it has not yet been demonstrated whether higher powers could have the same or better effects while maintaining treatment safety.

This prospective, non-randomized, double-blinded pilot study aims to evaluate and compare 1500 and 1000 mW laser powers with a 577 nm wavelength MLT in terms of IOP reduction following POAG during a short-term follow-up session. The secondary outcomes include evaluating and comparing the best-corrected visual acuity (BCVA), corneal changes (monitored with the corneal central thickness (CCT) and endothelial cell count (ECC)), topical medications required, and complications according to the randomized laser powers.

## 2. Materials and Methods

In this prospective, non-randomized, double-blinded pilot study, a group of consecutive patients with POAG at the Institute of Ophthalmology (University of Modena and Reggio Emilia, Italy) was included. All patients provided their written, informed consent. The study was approved by the local ethics committee (study number 1152/2019/DISP/AOUMO) and was conducted in accordance with the tenets of the Declaration of Helsinki. This trial was also registered (ClinicalTrials.gov number NCT 04900142).

Patient selection was based on the physical presence of a single collaborator (BB) who established patient eligibility and discussed the study with the patients. The criteria of inclusion specified a POAG diagnosis for patients who never received trabeculoplasty laser or glaucoma surgery intervention using one or more topical glaucoma medications and patient availability for extensive testing. Patients with a secondary glaucoma (neovascular, uveitic, or steroid-induced glaucomas), previous history of glaucoma surgery, severely decompensated glaucoma with IOP > 30 mmHg, those unable to collaborate during the execution of the laser procedure, and those with corneal diseases involving the endothelium (cornea guttata, Fuchs dystrophy) were excluded.

MLT with a 577 nm yellow wavelength (IQ 577^®^ Laser System model; IRIDEX, Mountain View, CA, USA) with an MLT gonioscopic lens (Iridex Corporation, Mountain View, CA, USA) was used. MLT at 1500 or 1000 mW were randomly assigned to an eye of each patient, with the second eye of each patient assigned to the non-randomized power. All other laser parameters remained the same (300 μm spot diameter, 300 ms duration, 15% duty cycle, and treatment extended to 360° of the trabecular meshwork). Treatments were performed with a single session of laser by the same expert physician (BB) with experience in the use of the yellow-wavelength MLT laser.

All patients underwent a complete ophthalmic examination at baseline (T0), including BCVA, IOP, CCT, and EEC, evaluating the number of applied topical medications for each patient.

The BCVA was evaluated with Early Treatment Diabetic Retinopathy Study (ETDRS) charts (Lighthouse Int., New York, NY, USA) at 4 m with assessments in letters. IOP was measured by the same expert operator (BB) with a Goldmann applanation tonometer (AT900D, Haag-Streit International, Koeniz, Switzerland) and calculated as the mean of 3 consecutive measurements. CCT and ECC were evaluated with SP-02 Perseus^®^ (CSO, Costruzione Strumenti Oftalmici, Scandicci, Firenze, Italy).

### MLT Technique

A drop of Pilocarpine 1% was selectively applied to the eyes with a poor view of the irido-corneal angle. Topical anesthesia was provided by the instillation of oxybuprocaine 0.4% eye drops preserved with p-hydroxybenzoate (Benoxinato Cloridrato, Alfa Intes, Naples, Italy), and a gonioscopy lens was applied. Treatment began in the lower sections of the eye, where the anatomical structures are often best identifiable. MLT treatment was standardized: 10 spots for every 30° for a total of 120 spots per eye, distributed over 360° of the trabecular meshwork. Bromfenac 0.09% eye drops twice/daily for one week were prescribed post-intervention in all cases.

Follow-up included IOP measurements one hour from the procedure (T1). If any treatments for glaucoma were required, they were generally continued until the following programmed visit. The day after the treatment (T2), a visit to the slit lamp was performed to evaluate any adverse effects, with repeated IOP assessments and CCT and ECC performed at 24 h. These examinations were repeated at one (T3), three (T4), and six months (T5) from the procedure. Both treating and visiting physicians were blinded to eye randomization.

Treatment was considered effective when the IOP reduction was >20% compared to the baseline values. At T3, topical medications for patients with a >20% IOP reduction were reassessed and, in patients using increased medications, one topical medication was removed.

A statistical analysis was performed using STATA14 (StataCorp. 2015. Stata Statistical Software: Release 14. College Station, TX, USA: StataCorp LP). Statistical analyses were conducted using the one-way analysis of variance and, in the comparison of the groups over time, the analysis of variance with the Bonferroni correction. A value of *p* ≤ 0.05 was considered statistically significant. The data, unless otherwise indicated, are presented as the mean ± standard deviation (SD).

## 3. Results

Eighteen eyes of nine patients (55.6% males) with POAG were enrolled in the study. The average age was 80.3 ± 6.2. The clinical baseline data of the POAG patients treated with MLT are presented in Table 1. Among the groups, there were no significant differences according to any clinical data. Hypotonic efficacy < 20% IOP was achieved for 14/18 eyes (77%), while in 2 patients the treatment was not effective. Comparative analyses of ECC, CCT, IOP, and BCVA, and topical medications for MLT 1500 and 1000 mV for each study time point did not reveal any statistical differences; observe Table 2. In particular, at 6 months follow-up, BCVA and CCT remained stable, while ECC was reduced; however, this occurred without any statistically significant values between pre- and post-treatment examinations or in the comparison between the two groups.

**Figure 1:**
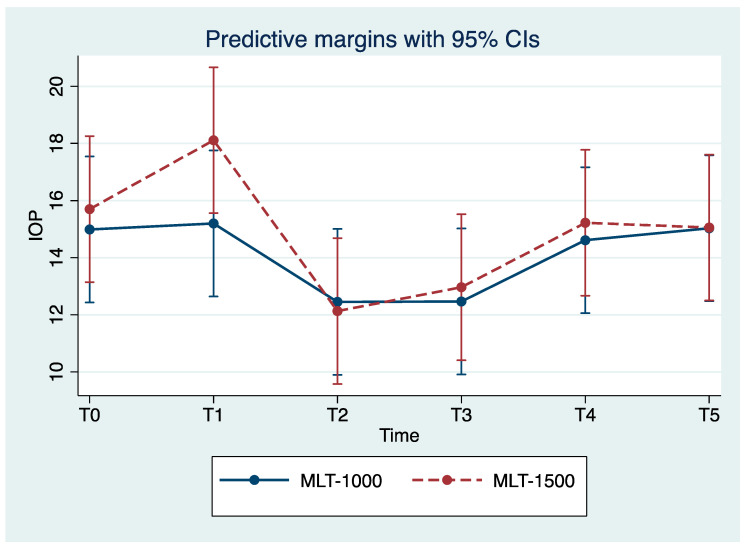
Mean intraocular pressure (IOP) at each study time point (mean ± SE). MLT, micropulse laser trabeculoplasty; IOP, intraocular pressure. T0, baseline; T1, treatment; T2, 24 h after treatment; T3, 1 month post-treatment; T4, 3 months post-treatment; T5, 6 months post-treatment.

We observed reduced IOP values for all eyes with the maximum reduction in IOP achieved for T2. Over the study period, the MLT 1500 group observed a significant improvement in IOP, with mean reduction values of 22.9% and 17.8% for T2 and T3 compared to the baseline values (*p* = 0.004, *p* = 0.024), respectively. Less pronounced improvements in IOP were observed among the MLT 1000 group, with a mean reduction of 17.3% for both T2 and T3, compared to the baseline values (*p* = 0.044, *p* = 0.012), respectively; observe Table 2 and Figure 1. For T4, IOP mean values returned close to those registered prior to the treatment and were stable for T5.

Complications were recorded in a single patient; a single, transient IOP spike of ~15 mmHg for T1 in the eye randomized to MLT 1500 and ~5 mmHg in the eye randomized to MLT 1000. There were no clinically visible corneal changes during the slit-lamp examination. There were no significant changes in the corneal parameters (CCT and ECC) registered during T4 and T5.

**Figure 2:**
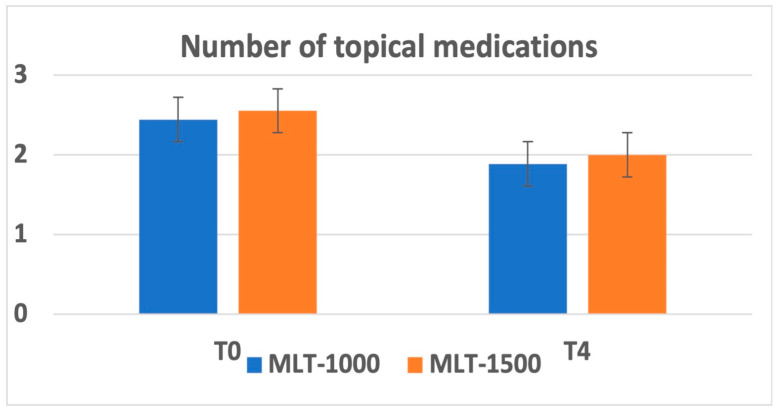
Number of topical medications at baseline (T0) and at 3 months after MLT intervention (T4).

Overall, there was a reduction in the number of topical medications administered at 1 month after treatment recorded in 7 patients and maintained during the follow-up. The average number of medications was reduced from 2.56 ± 1.13 to 2.00 ± 1.22 for the MLT 1500 group and from 2.44 ± 1.01 to 1.89 ± 1.05 for the MLT 1000 group. No significant differences were observed between the two groups; observe Figure 2.

## 4. Discussion

Our study shows that MLT with 577 nm for patients with POAGs can be performed effectively reducing IOP with either 1500 or 1000 mW. There were no significant differences among the two groups in terms of corneal changes (captured with ECC and CCT examinations), with a reduction in the overall topical medications administered and minimal complications over the post-intervention 6-month period.

Standard argon laser treatments (ALT and SLT) increased the aqueous outflow through the trabecular meshwork causing coagulative and structural damage with an increased inflammatory response and pigment dispersion frequently causing transient IOP spikes and ocular discomfort [10,11,12,13,14,15,16]. MLT has been recorded to stimulate a biological response without causing any macroscopic or microscopic changes in the trabecular meshwork, thereby inducing less inflammation and reducing tissue damage [24,25]; this was also confirmed by a histologic analysis performed on human corneoscleral rim tissue from cadaver eyes [26].

Moreover, the energy levels of laser trabeculoplasty with standard laser treatments (ALT and SLT) can be calibrated during treatment as microbubbles or where areas of whitening of the trabeculae are detected. With MLT, there are no clinically appreciable tissue changes or observable treatment endpoints [18,20,21]; therefore, the assessment of the adequacy of the parameters needs to be performed post-operatively, and the optimal power for MLT is still unclear.

Ingvoldstad et al. presented the first clinical results of MLT in 2005. In this pilot study, the authors performed MLT with an 810 nm diode laser at a power of 2000 mW (300 μm spot, 200 ms, 15% duty cycle over 360° TM) and reported significantly lower IOP results compared to the baseline, with a reduction of 18.3% in a 3-month follow-up, similar to the ALT technique, but with less inflammation [21]. However, Detry-Morel et al., comparing MLT with ALT using the same 810 nm laser, observed that MLT was still inferior to ALT in terms of its IOP-lowering effects in a mid-term follow-up session (12.2% decrease with MLT vs. 21.8% with ALT) [27].

The discordant results offered by the 810 nm laser led researchers to investigate novel techniques for MLT, and other studies investigated different parameters of MLT (laser wavelengths, power, spot diameter, and degrees of treatment) with varying results [28,29,30,31,32,33,34,35,36,37].

Recently, 532 and 577 nm lasers for MLT were investigated, since the 810 nm laser was thought to be not sufficiently effective in targeting pigmented trabecular meshwork cells. MLT with a 532 nm green wavelength (same wavelength of SLT) at 1000 mW was investigated, producing promising results [28,36,38]. However, the 577 nm yellow-wavelength laser is, at present, the most used laser for MLT, and a power of 1000 mW has been proven to be effective, with significant reductions in IOP at 6 months [20,37]. When compared to SLT, MLT was observed to be comparable in terms of its IOP-lowering effect with reduced IOP spikes in comparison to SLT [30,38], and, moreover, MLT was found to be better tolerated in terms of pain experienced during and after treatments [25]. Moreover, successful MLT treatment after previous SLT treatment has been reported, suggesting that this approach could also be effective in a pre-treated trabecular meshwork [28]. The most common spot dimension employed in MLT is 300 μm [29] and the best angle of treatment has been defined at 360° [20,35,37], as 180° is considered to provide inadequate treatment coverage [30,32]. Moreover, a recent paper showed that better results were reported when MLT treatment was conducted by a physician with previous MLT experience, underlining the importance of a steep learning curve in order to achieve optimal results with MLT [31].

Our study is the first to investigate the effect of a 1500 mW 577 nm MLT. Our study confirmed an overall IOP reduction, suggesting the efficacy of treatment with either 1500 or 1000 mW, with a greater IOP reduction in the 1500 mW group at 24 h (22.9% vs. 17.3% in the MLT 1000 group) and during the follow-up session; however, no significant differences were evident (see Figure 1, Table 2). Similar efficacy results suggest that the power of the laser does not significantly influence IOP reduction.

In our experience, the reduction in IOP achieved 24 h following treatment (T2) was in line with the results recently reported in the literature [28,39]. Makri et al. recently observed that 532 nm MLT at 1000 mW used for pseudo-exfoliative glaucomas did not result in any significant, potentially dangerous IOP spikes during the first 24 h [39]. We only observed a 1 h post-treatment IOP spike in one patient with higher pressure in the 1500 mW eye than in the 1000 mW eye. However, this complication was self-limited and IOP returned to lower values for the 24 h control without any treatment.

In our study, we observed that IOP values returned to baseline at the three-month follow-up period and remained stable at six months, and this may have been due to the reduced laser-induced anti-inflammatory effects three months after the treatment. We assumed that, in some patients, treatment could be performed again to maintain the results over time. However, we registered a reduction in the number of topical medications assumed 1 month after the treatment, and this was in line with two recent studies reporting a statistically significant reduction in the number of topical medications administered for either MLT or SLT [37]. This result highlights the importance of MLT treatment in improving the quality of life of patients by reducing the number of topical medications, thereby reducing the topical drugs burden and side effects in relation to time and cost.

Finally, the safety of MLT treatment on corneal morphology was also confirmed. The trabecular meshwork is close to the cornea; therefore, the corneal tissue can be accidentally damaged by the laser. Several investigations reported corneal alterations following ALT and SLT laser procedures [15,40,41,42,43,44,45]. A previous investigation reported the safety of MLT treatment using a 1000 mW 532 nm laser for a single session [36]. There are no studies, at present, investigating the corneal parameters following 577 nm MLT. In our study, we did not obtain any statistically significant differences between the two groups in terms of corneal thickness or endothelial microscopy during a 6-month follow-up period, demonstrating that either 1000 or 1500 mW can be safely used without causing any damage to the corneal endothelium.

This pilot study was limited by the inclusion of a small sample size, including a heterogeneous patient cohort (assuming different topical medications), with relatively older-aged patients than those included in other similar investigations, and a relatively short follow-up period. Furthermore, a control group was not included, which could have measured the effectiveness of the laser compared to any other medical treatment.

## 5. Conclusions

In conclusion, MLT for POAG with a 577 nm laser at either 1500 or 1000 mw lowered the IOP following treatment with a similar hypotonic efficacy. This treatment modality resulted in a reduction in topical medications being of greater importance in improving the quality of life of patients, reducing drugs burden and side effects in a time- and cost-effective manner. The treatment did not induce alterations in the corneal endothelium, demonstrating that either power setting can be performed safely. Further studies are needed to confirm our results to exploit the MLT technique by clarifying the optimal duration of the treatment and its repeatability over time, thus defining the role of MLT in the management of PAOG patients.

## Figures and Tables

**Table 1:** Clinical baseline values of eyes randomly assigned to yellow-wavelength micropulse laser trabeculoplasty at 1500 or 1000 mW. MLT, micropulse laser trabeculoplasty; BCVA, best-corrected visual acuity (ETDRS letters); ECC, endothelial cell count (cell/mm^2^); CCT, central corneal thickness (microns); IOP, intraocular pressure (mmHg).

	MLT 1500	MLT 1000	*p*-Value
*Eyes*, *n*	9	9	
*BCVA*, *letters (±SD)*	42.9 (±17.2)	49.3 (±5.1)	0.297
*ECC*, *cell*/mm^2^*(±SD)*	2271 (±461)	2143.6 (±445.2)	0.559
*CCT*, μm *(±SD)*	525.7 (±24.2)	533.3 (±31.4)	0.575
*IOP*, mmHg *(±SD)*	15.7 (±4.8)	14.9 (±3.4)	0.724
*N. of glaucoma topical medications*	2.5 (±1.1)	2.4 (±1.0)	0.829

**Table 2:** Clinical data for each study time point according to micropulse laser trabeculoplasty at 1500 or 1000 mW. ECC, endothelial cell count; CCT, central corneal thickness; BCVA, best-corrected visual acuity; IOP, intraocular pressure. T0, baseline; T1, treatment; T2, 24 h after treatment; T3, 1month post-treatment; T4, 3 months post-treatment; T5, 6 months post-treatment.

		MLT 1500	MLT 1000	*p*-Value
**ECC, cell/mm^2^ (±SD)**	T0	2271 ± 461	2143 ± 445	0.559
	T1	2218 ± 474	2121 ± 446	0.660
	T4	2157 ± 463	2003 ± 470	0.494
	T5	2179 ± 424	2071 ± 506	0.460
**CCT, μm *(±SD)***	T0	525 ± 24	533 ± 31	0.575
	T1	531 ± 28	538 ± 29	0.601
	T4	529 ± 27	536 ± 32	0.488
	T5	528 ± 28	539 ± 31	0.087
**IOP, (mm Hg)**	T0	15.7 ± 4.8	14.9 ± 3.4	0.724
	T1	18.1 ± 7	15.2 ± 3.5	0.282
	T2	12.1 ± 3.8	12.4 ± 2.6	0.837
	T3	12.9 ± 2.9	12.4 ± 2.9	0.725
	T4	15.2 ± 3.3	14.6 ± 3.6	0.691
	T5	15.0 ± 2.9	15.0 ± 3.1	0.987
**BCVA, (ETDRS chart letters)**	T0	42.9 ± 17.2	49.3 ± 5.1	0.297
	T5	42.1 ± 16.9	49.6 ± 4.9	0.132
**Medications, n *(±SD)***	T0	2.5 ± 1.1	2.4 ± 1.0	0.829
	T3/T5	2.0 ± 1.2	1.9 ± 1.0	0.839

## Data Availability

All the data will be available upon request from the corresponding authors.
